# An isolated white‐tailed deer (*Odocoileus virginianus*) population on St. John, US Virgin Islands shows low inbreeding and comparable heterozygosity to other larger populations

**DOI:** 10.1002/ece3.7230

**Published:** 2021-02-03

**Authors:** Suzanne L. Nelson, Scott A. Taylor, Jon D. Reuter

**Affiliations:** ^1^ Department of Integrative Physiology University of Colorado Boulder Boulder CO USA; ^2^ Department of Ecology and Evolutionary Biology University of Colorado Boulder Boulder CO USA; ^3^ Office of Animal Resources Department of Psychology and Neuroscience University of Colorado Boulder Boulder CO USA

**Keywords:** deer, genetics, invasive, island, *Odocoileus virginianus*, St. John, white‐tailed deer

## Abstract

This is the first study to document the genetic diversity of the white‐tailed deer population on St. John, US Virgin Islands. The island population was founded by a small number of animals, has very limited hunting or predation, and recently experienced a reduction in size following an extended drought in 2015. DNA samples were collected from hair from 23 anesthetized adult deer (13 males, 10 females) ranging in age from 1 to 8 years (3.36 ± 1.9 years) and also from fecal DNA samples, for a total of 42 individuals analyzed for genetic diversity. The St. John deer data set averaged 4.19 alleles per marker and demonstrates the second lowest number of alleles (A) when compared to other populations of *Odocoileus virginianus* (4.19). Heterozygosity was similar to the other studies (0.54) with little evidence of inbreeding. To explain the level of heterozygosity and level of inbreeding within the St. John population, three hypotheses are proposed, including the effect of intrinsic biological traits within the population, a recent infusion of highly heterogeneous loci from North American populations, and a consistent level of immigration from a nearby island. Additional work is needed to further understand the genetic history of the St. John and regional deer populations.

## Graphical abstract

This is the first study of the genetic profile for the white‐tailed deer population of St. John, Virgin Islands, which was introduced with a very small founder population over 200 years ago. The results show high levels of heterozygosity for an isolated island population. Several ideas for the degree of heterozygosity are proposed.

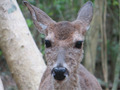

## 1 INTRODUCTION

Low levels of genetic diversity can result from many factors, including small founder populations, stochastic events that reduce population size, and inbreeding (Markert et al., 2010; Pekkala et al., 2012; Trinkel et al., 2010). Small and inbred populations can exhibit negative consequences for growth, disease resistance, survival, fertility, and development (Ruin‐Lopez et al., 2012; Spielman et al., 2004), which can further diminish population size over time and lead to increased extinction risk (Reed & Frankham, 2003). The deleterious effects of inbreeding depression may be more pronounced under stressful conditions to the population than in benign conditions and result in conditionally expressed deleterious genes (Bouzat, 2010; Fox & Reed, 2010). To increase genetic diversity, new individuals from outbred populations can be added to the population and increase genomic heterozygosity. This can be highly effective in reducing the deleterious effects of inbreeding to a population (Fredrickson et al., 2007; Heber et al., 2012).

Island populations are often highly isolated spatially and are more prone to losing genetic diversity through genetic drift, or by bottlenecks due to small population sizes at the population's founding (Jamieson, 2007). However, islands offer several advantages to examining genetic processes. Immigration and emigration are often minimal, and therefore, selection and genetic drift become more dominant as the processes most likely to affect levels of genetic variation (Pemberson et al., 1996). In addition, island populations tend to be more tractable because they are restricted within the physical confines of the island perimeter. As a result, the genetic composition of an island population is highly influenced by the number of founding individuals, their genetic diversity, the population rate of increase over time, and the extent of gene flow within the population (Freeland, 2005; Simpson et al., 2013).

The deer of St. John, U.S. Virgin Islands have a unique history. The first mention of white‐tailed deer (*Odocoileus virginianus*) in the U.S. Virgin Islands was from a Danish ship log, where the captain mentions five white‐tailed deer being released on St. Croix during or before 1790 (Heffelfinger, 2011), most probably for hunting purposes. Deer were described as inhabiting the mountainous parts of St. Croix in 1840 (Seaman, 1966), and in 1854, some of the deer were moved to St. Thomas and subsequently swam to inhabit St. John (Heffelfinger, 2011). Additional deer were brought to St. Thomas and St. John from Texas and the Carolinas in the 1950s as part of a USDA translocation program (Baker, 1984). Reports of deer swimming between the islands of St. Thomas and St. John are quite consistent through time (Heffelfinger, 2011). Before an extended drought in 2015, the population was estimated at approximately 2000 deer on St. John. The deer are protected from hunting within Virgin Islands National Park, and there are no natural predators on St. John. The deer are highly habituated to humans and show very limited fear as they forage near popular tourist trails and beaches during daylight hours. The deer are not actively managed by the Virgin Islands National Park for a targeted density. They are very popular with tourists, and residents consider them natural fauna because they have been present on the island for hundreds of years. As an introduced species, they have a significant impact on island flora and native plants. One of our research goals was to determine a sustainable density for the deer on St. John to remain below carrying capacity for the island.

The size of the deer population on St. John changes in response to environmental conditions and food availability. An increase in of twinning is often a sign that food is abundant and that the population is increasing (DeYoung, 2011). Signs of a stressed and a decreasing population include high levels of mange and tick infestation, as well as muscle atrophy and poor body condition (Nemeth et al., 2013; Nelson et al., 2017). More recently, the St. John deer have undergone the intense stress of two category 5 hurricanes, Irma and Maria, in the fall of 2017. The current population estimate of deer on St. John following the two recent hurricanes is unknown.

The objective of this study was to determine the level of inbreeding in this isolated population of white‐tailed deer on St. John following a drought on the island. A formal study of the genetics of this group has not been previously conducted, and the level of heterozygosity for this population has yet to be described.

## 2 METHODS

**FIGURE 1:**
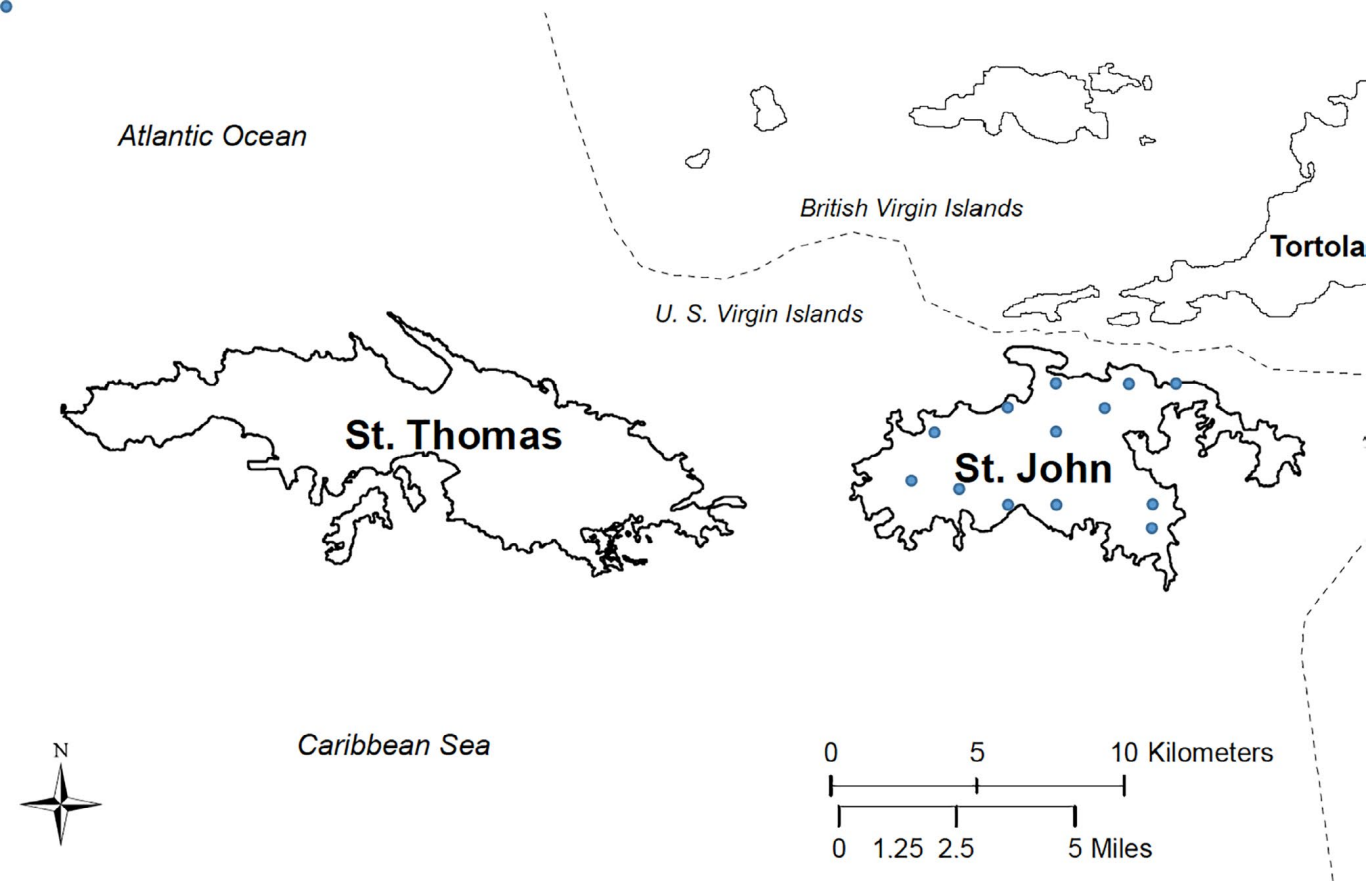
Spatial orientation of St. Thomas (left), and St. John (right), US Virgin Islands. Blue dots on St. John indicate where genetic samples were collected

St. John is part of the US Virgin Islands which includes St. John, St. Thomas, St. Croix, and Water Island. St. Thomas is the nearest island which also contains deer (Figure 1). Virgin Islands National (VINP) park lies on the island of St. John and comprises 60% of the landmass of the island. VINP protects one of the largest and most mature tracts of secondary dry forest in the eastern Caribbean (Ray et al., 1998). The island vegetation is largely represented by low‐to‐mid elevation dry scrub forest on soils with fairly low soil nutrient content (Ostwalt et al., 2006) and is considered marginal habitat for deer. A severe drought was present on St. John and the surrounding region that lasted for the duration of 2015 and caused water, food, and environmental stress to the St. John deer population (Nelson et al., 2017). During the drought, deer showed signs of stress such as highly elevated tick and mange levels, muscular atrophy, poor coat quality, weight loss, lethargy, reduced reproduction, and death (Reuter & Nelson, 2018).

DNA samples were collected in two ways, either by collecting hair samples from deer while they were anesthetized or by collecting DNA from fecal samples. The data were collected within three separate field site visits, all within the years 2015–2016. Sample collection was restricted to certain areas because of the availability of access trails, but represents major portions of the small island (Figure 1). To collect hair samples, adult deer were anesthetized using butorphanol, azaperone, and medetomidine (BAM, Wildlife Pharmaceuticals). Only adult does and bucks were immobilized for this project, no pregnant deer or fawns were used. Vitals monitored included heart rate, respiratory rate, mucous membrane color, body temperature, time to recumbency, and recovery. Following hair collection and after examination, the anesthesia was reversed with 2–3 ml of atipamezole (25 mg/ml) and 0.5 ml of naltrexone (50 mg/ml, Wildlife Pharmaceuticals). Deer recovered to standing with full stability within five minutes (Reuter & Nelson, 2018). Hair samples were individually labeled and placed in coin envelopes in frozen storage until analysis.

Fecal DNA samples were collected only from freshly deposited fecal samples with the deer in view. Several toothpicks were rubbed gently over the surface of the fecal sample for each sample collected. The samples were allowed to dry and placed in a coin envelopes in frozen storage until analysis. Research on live animals followed ASM guidelines (Sikes et al., 2016) and was completed under Scientific Research and Collection permit VIIS‐2016‐SCI‐0026 for the USVI National Park and the University of Colorado Boulder and the National Park Service Institutional Animal Care and Use Committee (1602.01‐15Mar2016).

DNA was extracted from both the hair samples using QIAGEN DNeasy Blood and Tissue kits and following QIAGEN’s tissue protocol. All hair samples yielded at least 10 guard hair roots (Paetkau, 2003). A standard set of 21 microsatellite markers that are used for parentage certification in game farming applications, and which were found originally in populations of mainland deer from North Carolina, Wisconsin, and Texas, were amplified for the 80 adult deer sampled on St. John (Wildlife Genetics International, Inc.).

Individuals with > 10 genotyped microsatellites (*N* = 42, all 21 microsatellites amplified for 23 individuals) were analyzed using GENEPOP (Ver. 4.2) (Raymond & Rousset, 1995; Rousset, 2008) to determine number of alleles per locus, observed heterozygosity, and inbreeding coefficient (*F*_IS_) for comparison to non‐island populations of white‐tailed deer.

## 3 RESULTS

For DNA samples collected from hair, a total of 23 adult deer (13 males, 10 females) were sampled, ranging from 1 years to 8 years old (3.36 ± 1.9 years) on the basis of a palpated tooth shape (Reuter & Nelson, 2018). Anesthesia was uncomplicated, with no observed injuries or capture myopathy. After anesthetic reversal, the deer recovered quickly; most were ambulatory within 5 min. All deer appeared healthy and robust following the capture session. For DNA samples collected from feces, 56 samples were used for this analysis. However, only 42 individuals had data for at least 10 microsatellites (max 21, *N* = 23) from the entire data set and were used in the final analysis. Some of the fecal samples came from the same individuals, which further reduced our sample from 80 down to 42.

When compared to other populations of *O. virginianus* from the continental United States, as well as Canada and Mexico, the deer population of St. John possesses the second lowest allelic richness (*A*) of all of the comparison populations compared (Table 1), but observed heterozygosity is similar to other populations (0.54) (Table 1). There is little evidence of inbreeding in the St. John population of white‐tailed deer—the F_IS_ value does not differ significantly from zero.

**TABLE 1:** Results from the St. John deer population as compared for other published studies from the continental United States, Canada, and Mexico

Location Subspecies	Genetic marker	*N*	*A*	*H*_O_	*F*_IS_	Reference
U.S. Virgin Islands *Odocoileus virginianus*	>10 (max 21) microsatellites	42	4.19	0.54	0.05	This study
Mexico						
*O. v. texanus*	12 microsatellites	39	11.9	0.53	0.38	De la Rosa‐Reyna et al. (2012)
*O. v. carminus*		12	7.2	0.64	0.19	
*O. v. veraecrucis*		20	7.8	0.59	0.23	
*O. v. sinaloae*		3	3.1	0.61	0.06	
*O. v. yucatanensis*		16	5.5	0.41	0.42	
U.S. Pacific coast						
*O. v. leucurus*	16 microsatellites	124	6.2	0.44	—	Hopken et al. (2015)
*O. v. ochrourus*		74	6.4	0.5	—	
USA Wisconsin/Iowa	12 microsatellites	249	12.7	—	0.03	Lang and Blanchong (2012)
USA Mississippi	17 microsatellites	543	5.93	0.71	0.06	DeYoung et al. (2003)
Canada (British Columbia, Alberta, Saskatchewan)	14 microsatellites	1,960	13.1	0.66	0.04	Cullingham et al. (2011)

Note

*N* = number of individuals, *A* = allelic richness, *H*_o_ = observed heterozygosity, *F*_IS_ = inbreeding coefficient.

## 4 DISCUSSION

This is the first study to document the genetic diversity of the white‐tailed deer population on St. John. This population is characterized by a small number of animals in its founder population, a lack of hunting or predation, and a recent extended drought. Despite these factors, the levels of heterozygosity for this population were comparable to mainland populations and there was little or no evidence of inbreeding. We propose three potential hypotheses in an attempt to explain the level of heterozygosity currently seen within the St. John deer population.

Hypothesis 1 There may be intrinsic biological traits of the species, including the potential for rapid population growth and iteroparity that alter the expected outcome for genetic loss.

The potential for rapid population growth due to high reproductive success may have reduced the overall genetic loss to the St. John population. For example, when released into the forests of St. John upon their first introduction to the island, the deer experienced an ecological open niche free from predators and increased rapidly (Seaman, 1966). Despite its small founder population, the St. John deer population spent a relatively short time period at a small size (Heffelfinger, 2011). This may have allowed the population to largely retain its genetic diversity because fast population growth minimizes loss of genetic diversity, assuming high survival and reproductive success (Kekkonen et al., 2012; Murphy et al., 2015). Thus, the natural history parameters within deer that allow for high reproductive rates (e.g., twinning is common and triplets occur with excellent maternal nutrition) may have altered the genetics of the group over time, particularly within an environment of with low competition and high food availability. The second infusion of genes into the population, with the USDA translocated deer in the 1950s (Baker, 1984; Heffelfinger, 2011), may have increased the deer genetic heterozygosity further, but might play a more minor role than expected because of the allelic retention following the initial rapid population growth upon their introduction to the island (Kekkonen et al., 2012).

In addition to the biological potential for rapid population growth for deer, iteroparity, resulting in overlapping generations, may have also influenced heterozygosity of the St. John deer (Murphy et al., 2015). In species with overlapping generations, allelic drift can be lower than in species without overlapping generations (Kekkonen et al., 2012). This has been found to be particularly true for individual‐based population genetic models rather than classic population genetics models (Pemberson et al., 1996). Together, rapid population growth and iteroparity may have had an additive effect in retaining heterozygosity within the population, resulting in higher allelic reserves than would be predicted for an isolated island population of deer on St. John for more than 200 years.

Hypothesis 2 The deer of St. John may have high levels of genetic diversity due to an infusion of heterogeneous loci in the recent past.

White‐tailed deer are one of the most abundant of all New World deer species, and one that enjoys a world‐wide distribution (Heffelfinger, 2011). Due to this vast geographic distribution, phenotypic and genotypic variations exist throughout their range due to either isolation, phenotypic plasticity, and/or adaptations to local habitat, forage, and climactic conditions, resulting in 38 recognized subspecies (Heffelfinger, 2011; Strickland & Demarais, 2008). In addition, white‐tailed deer have been the been part of domestic and international restoration and translocation programs that have further increased their allelic diversity globally. For example, the deer in the state of Virginia were restocked from deer in eleven separate states, and each state received hundreds of deer from Wisconsin as part of restocking programs (Matchington et al., 1995). Furthermore, the number of alleles per locus for white‐tailed deer was found to be significantly different from those of mammals in general (Breshears et al., 1988), further influencing the genetic architecture of deer populations.

The history of the deer on St. John states that additional deer were brought to St. Thomas and St. John from Texas and the Carolinas in the 1950s as part of a USDA translocation program (Baker, 1984; Heffelfinger, 2011). Both the Texas and Carolina deer populations are noteworthy in their levels of genetic heterogeneity either due to geography or isolation (Erickson, 1979; Hillestad, 1984). In addition to the Carolina and Texas deer populations being restocked, the Texas population has not experienced any kind of a population bottleneck that reduced its allelic diversity (Erickson, 1979; Rhodes & Smith, 1992) resulting in a highly genetically diverse source population. Genetic heterogeneity among deer populations is generated over short geographical distances, sometimes as little as 5 km (Sheffield et al., 1985), which is unexpected for this large and highly mobile species (Smith et al., 2001). As a result, a high degree of genetic heterozygosity found within deer populations translates into differences in disease resistance and immune response among deer subspecies (Gaydos et al., 2002; Johnson et al., 2006).

Therefore, perhaps the genetic diversity of the St. John deer after approximately 200 years is not as low as expected due to the influx of highly heterogeneous alleles that came from the infusion of deer from source populations in Texas and the Carolinas in the 1950s. This diversity, coupled with the short duration of time since their infusion into the current population, (approximately 70 years), may have added a significant amount of genetic diversity of deer currently living on St. John.

Hypothesis 3 A consistent level of immigration from St. Thomas may have resulted in genetic rescue to the St. John deer population.

Many species on islands or within small isolated populations that experience bottlenecks can exhibit the effects of inbreeding depression and the subsequent loss of genetic and allelic variation (Kekkonen et al., 2012). These populations often require genetic contributions from unrelated individuals to reduce their number of deleterious alleles, a process called genetic rescue (Fredrickson et al., 2007; Tallman et al., 2004). Genetic rescue can have a significant effect on fitness, including increases to composite fitness, which combines fecundity and survival estimates (Frankam, 2015). Additionally, the effects of genetic rescue tend to be most pronounced in animals living within stressful environments (Frankham, 2008). Outbred individuals with increased genetic diversity demonstrate increased resilience through juvenile survival, sperm quality, and immunocompetence compared with inbred control individuals, even if the genetic rescue donors were from another inbred population (Fredrickson et al., 2007; Heber et al., 2012) that contained low genetic variation and fixed deleterious alleles (Vila et al., 2003; Kekkonen et al., 2012). Genetic rescue is most successful within a population if the novel alleles continue in subsequent generations and can potentially influence lifetime reproductive success for individuals within a population (Fredrickson et al., 2007; Heber et al., 2012).

The deer of St. John could have possibly benefitted from genetic rescue, resulting in their current level of heterozygosity. Despite the 6.4 km of open water and challenging currents, deer have been consistently described and observed swimming between the islands throughout their history, and most often from St. Thomas to St. John (Heffelfinger, 2011). The deer on St. Thomas are considered agricultural pests and have been actively hunted. Hunting may have resulted in different selective pressures that altered the genetic base of the St. Thomas deer. The introduction of new genes from St. Thomas, even though they share a similar history, may be enough to diversify the gene pool of the St. John deer and maintain healthy heterozygosity levels. It is currently not known what number of deer are immigrating from St. Thomas to St. John. Also, there has been no study of the genetics of the St. Thomas deer to know their current levels of allelic diversity. However, the allelic contribution of the St. Thomas deer to the St. John population may be significant over time and may have acted as a steady infusion of new alleles to the population, even if the deer population of St. Thomas is not genetically very diverse.

There have been changes to the deer population of St. John compared to mainland deer as a result of their isolation on the island of St. John for over 200 years. In the absence of predation, these changes appear to be largely environmentally induced. The individual and population changes observed in the St. John deer population include reduced physical stature of the deer (Heffelfinger, 2011; Reuter & Nelson, 2018; Webb & Nellis, 1981), high levels of disease manifestation for ticks and mange (Nelson et al., 2017), acute die‐offs resulting from epizootic hemorrhagic disease virus (EDHV) (Reuter & Nelson, 2018), and reduced fecundity levels observed in the deer on island. Many of these changes may be multifaceted in origin. For example, reduced physical stature could be influenced by genetics (Webb & Nellis, 1981), nutritional deficiencies (Hewitt, 2011), food scarcity (Robbins, 2012), phenotypic plasticity (Rozzi & Lomolino, 2017), changes to climate (Gardner et al., 2011), or Foster's rule, where large mammals become smaller on islands through time (Foster, 1964; Millien, 2011). The drought in 2015 resulted in significant food and water stress to the deer population of St. John, resulting in a diminished number of deer (Nelson et al., 2017). Although episodic, stressful events like drought and hurricanes could be acting as strong evolutionary forces to the population and influence the genetic portrait of the population over time.

It is currently unknown which of the three proposed hypotheses explain the levels of heterozygosity found within the St. John deer population, or if the answer is a combination of several of the scenarios described. To identify the mechanism(s) responsible for preserving allelic diversity with more precision will require additional research and should include a genetic analysis of the source populations, a better understanding of the St. Thomas deer genetic profile, a larger sample of deer sampled, more detail on the history of deer introductions to both St. Thomas and St. John, and a more comprehensive approach to genetic data collection regionally. This paper represents a first study of the genetic composition of this population and is one is a series of papers describing the characteristics of this population of introduced deer to St. John (Nelson et al., 2017; Reuter & Nelson, 2018). This work can provide an important foundation for management decisions by resource managers on island and inform future management actions by Virgin Islands National Park to better manage the deer population within the carrying capacity of St. John. Overall, the deer of St. John provide an engaging case study to examine complex themes within ecology, including island ecology, predator‐free landscapes, isolated population dynamics, the founder effect, and the effects of episodic environmental stressors on both population dynamics and to individual animals.

## CONFLICT OF INTEREST

None declared.

## AUTHOR CONTRIBUTIONS

**Suzanne L. Nelson:** Conceptualization (lead); data curation (lead); funding acquisition (lead); investigation (lead); methodology (lead); writing‐original draft (lead). **Scott A. Taylor:** Formal analysis (lead); methodology (lead); software (lead); validation (lead). **Jon D. Reuter:** Data curation (lead); investigation (lead); methodology (lead); writing‐review & editing (supporting).
